# Comparison of matrix metallopeptidase-9 expression following cyclosporine and diquafosol treatment in dry eye

**DOI:** 10.1080/07853890.2023.2228192

**Published:** 2023-06-24

**Authors:** Ha Rim So, Jiwon Baek, Ji Young Lee, Hyun Seung Kim, Man Soo Kim, Eun Chul Kim

**Affiliations:** a Department of Ophthalmology, Bucheon St. Mary’s Hospital, College of Medicine, The Catholic University of Korea, Gyeonggi-do, Korea; b Department of Ophthalmology, Daejeon St. Mary’s Hospital, College of Medicine, The Catholic University of Korea, Daejeon, Korea; c Department of Ophthalmology, Seoul St. Mary’s Hospital, College of Medicine, The Catholic University of Korea, Seoul, Korea; d Department of Ophthalmology, College of Medicine, The Catholic University of Korea, Seoul, Korea

**Keywords:** Dry eye disease, MMP-9, 0.05% cyclosporin A, 3.0% diquafosol tetrasodium

## Abstract

**Purpose:**

We sought to evaluate the expression of matrix metalloproteinase-9 (MMP-9) in dry eyes treated with 0.05% cyclosporin A and 3.0% diquafosol tetrasodium.

**Methods:**

One-hundred ninety-five eyes of 195 patients with dry eye were divided into three groups as follows: group 1, cyclosporin group (*n* = 69); group 2, diquafosol group (*n* = 59); and group 3, artificial tears eyes (*n* = 67). All eyes were treated and followed up for three months. Schirmer I Test, corneal staining, tear-film break-up time (TBUT), and tear-film MMP-9 content were measured at three months and compared between groups. The expression of MMP-9 was confirmed using a point-of-care test device (InflammaDry®; RPS Diagnostics, Sarasota, FL, USA) and graded as zero to four points.

**Results:**

At the third month, MMP-9 expression was lower in group 1 as compared with in groups 2 and 3 (*p =* 0.020 and 0.006, respectively). The mean MMP-9 grade according to point-of-care testing was also lower in group 1 than in groups 2 or 3 (*p =* 0.002 and 0.038, respectively). MMP-9 showed a correlation with corneal staining in both groups 1 and 2 (all *p* < 0.001) and with Schirmer I Test and TBUT in group 1 (*p* = 0.018 and 0.015, respectively).

**Conclusions:**

MMP-9 expression and grade were lower after treatment with cyclosporin than after treatment with diquafosol in the dry eye disease. Anti-inflammatory treatment can decrease ocular MMP-9 levels in dry eye disease.

## KEY MESSAGES

MMP-9 expression and grade were lower after treatment with cyclosporin than after treatment with diquafosol in the dry eye disease. Anti-inflammatory treatment can decrease ocular MMP-9 levels in dry eye disease.

## Introduction

The definition of dry eye proposed by the Dry Eye Workshop states that it is a multifactorial disorder that can be classified as either aqueous-deficient or excessive evaporation [1]. Recent observations demonstrate that, in dry eye pathogenesis, an important role is played by both inflammation and apoptosis [2,3].

Matrix metalloproteinases (MMPs) are among the molecules released during the inflammatory process of dry eye. These are a family of zinc- and calcium-dependent enzymes involved in the breakdown of extracellular matrix in several physiological and pathologic processes [4,5]. It had been reported that MMPs play a fundamental role in extracellular matrix remodeling after wounding of the corneal surface. MMPs has been implicated in the pathogenesis of dry eye and blepharitis as well as other corneal surface conditions including allergic conjunctivitis, burns, sterile corneal ulceration, conjunctivochalasis, and pterygium [4,6–9]. Among the MMP family, MMP-9 (gelatinase B) is produced by the corneal epithelium, fibroblasts, infiltrating leucocytes, and the lacrimal gland [4,6,10,11].

MMP-9 is conventionally measured in the laboratory by enzyme-linked immunosorbent assay, multiplex bead analysis, and/or proteomic technology [6,12,13]. However, the recent introduction of a point-of-care MMP-9 immunoassay tool (InflammaDry®; RPS Diagnostics, Sarasota, FL, USA) enabled easy and fast measurement of MMP-9 in the office with high sensitivity and specificity [14,15].

Topical 0.05% cyclosporin A, 3.0% diquafosol tetrasodium, artificial tears are the most commonly used medications in dry eye treatment. The mechanism of action on dry eye is different among the three drugs. Cyclosporin A is known to reduce surface inflammation in dry eye by inhibiting T-cell activation and downregulating the production of inflammatory cytokines [16,17]. Conversely, diquafosol tetrasodium is a purinergic P2Y2 receptor agonist that stimulates water and mucin secretion from conjunctival epithelial cells and goblet cells [18,19]. Artificial tears are eyedrops used to lubricate dry eyes and help maintain moisture on the outer surface of eyes. The purpose of this study was to compare the expression of MMP-9 in dry eyes treated with either cyclosporin or diquafosol and their association with other dry eye markers.

## Methods

This retrospective, interventional, and comparative study was performed at the Department of Ophthalmology in Bucheon St. Mary’s Hospital at the Catholic University of Korea in Gyeonggi-do, Korea. As a retrospective study, we reviewed the medical records of patients treating dry eye syndrome with cyclosporin A, diquafosol, or artificial tears. The data provided to us excluded all personal identifiers. Approval for the retrospective analysis of these data was obtained from our Institutional Review Board, and the study was performed in accordance with the tenets of the Declaration of Helsinki. The Institutional Review Board (IRB)/Ethics Committee of Bucheon St. Mary Hospital approved this study protocol (XC16MIMV0056H) and waived the requirement for in-formed consent because of the retrospective nature of the study.

### Participants

Diagnosis of patient ‘Dry eye syndrome; dry eye; Keratoconjuncrtivitis sicca’ and prescription code ‘artificial tears, diquafosol, cyclosporine A’ were used to search the data for the past 2 years. Inclusion criteria were patients with primary aqueous-deficient dry eye whose disease and therapy regimen, including systemic as well as topical, were stable for at least one month before inclusion in the study. The grading of dry eye severity followed the 2007 International Dry Eye Workshop criteria [1]. In this study, level 2–4 were considered to be moderate to severe: significant subjective symptoms (e.g. scratchy or sandy sensation, itching, burning, redness, and photosensitivity), variable or marked central corneal staining, and Schirmer I Test results of 10 mm or less.

Patients were treated with cyclosporin A(Restasis®; Allergan Inc, Irvine, CA, USA), diquafosol(Diquas®; Santen Pharmaceutical Co Ltd., Osaka, Japan) and artificial tears as part of routine care, which was decided by the treating doctor and not for purpose of study. Patients were divided into three groups: group 1 was treated with 0.05% cyclosporin A two times per a day for three months; group 2 was treated with 3.0% diquafosol six times per a day for three months; and group 3 was treated with artificial tears. In addition to their individual group regimens, all patients were additionally treated with 0.1% sodium hyaluronate artificial tears (Tearinfree®; DHP Inc., Seoul, Korea) several times a day as needed. All patients were provided with guidance for using eyedrops at each visit and followed strictly to the guidance.

### Clinical assessment

All patients underwent a full ophthalmological examination, including corrected visual acuities, intraocular pressure, and slit-lamp examination. ST with topical anesthesia and corneal staining using fluorescein strips (fluorescein paper; Haag-Streit AG, Köniz, Switzerland) were performed at baseline. The corneal staining score was classified as zero to five points, based on the Oxford scheme [20]. Tear-film break-up time (TBUT) test, Schirmer I Test, corneal staining, Ocular Surface Disease Index (OSDI) score, and MMP-9 content were measured after three months of treatment. TBUT was also measured using corneal topography (Keratograph 5 M®; Oculus GmbH, Wetzlar, Germany).

### Measurement of MMP-9

**Figure 1.**
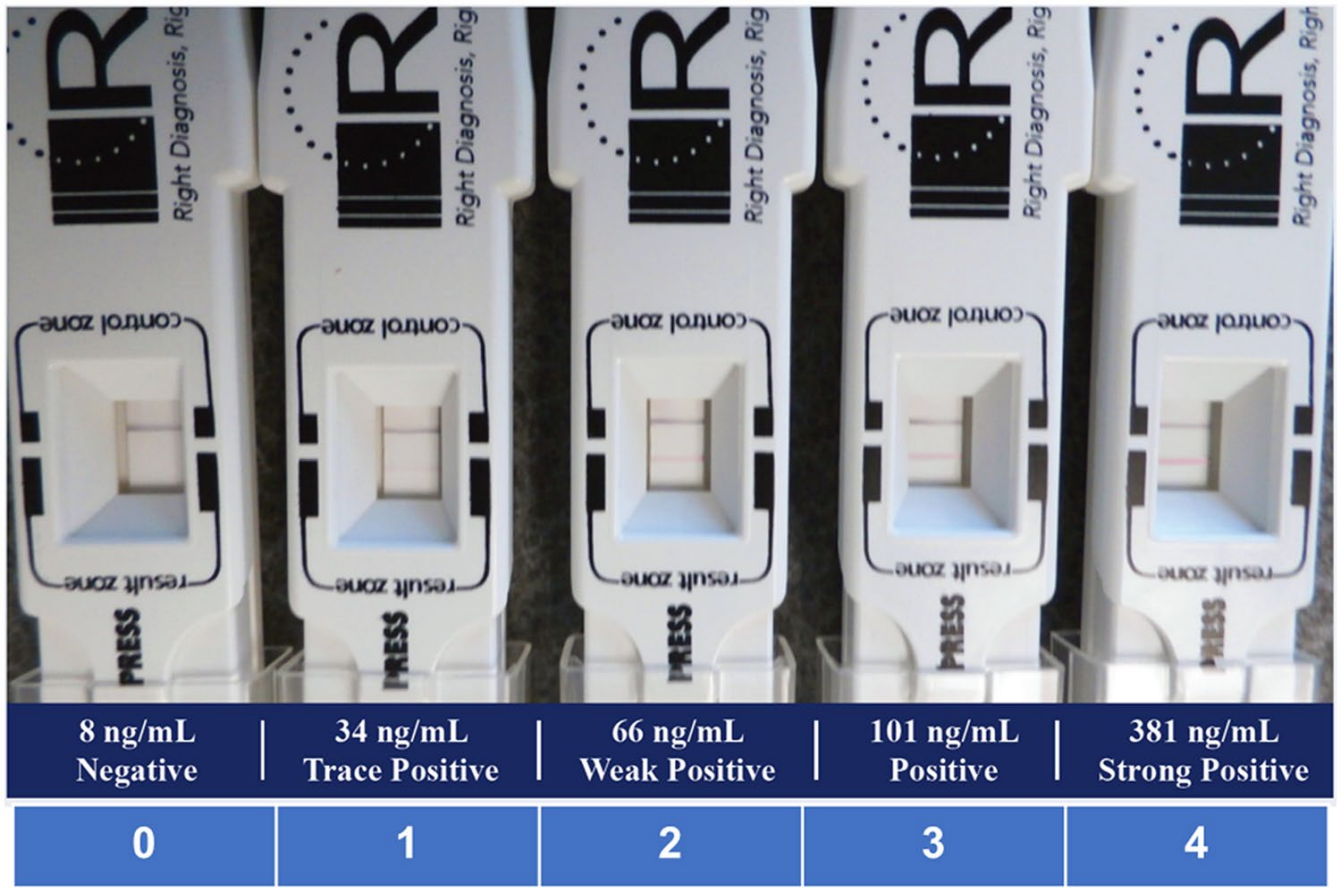
Reference image for MMP-9 grading. Semiquantitative interpretation using color intensity of the result line was done by assigning zero to four points according to the above reference image.

The InflammaDry® test was performed according to the package insert. In short, the sampling fleece was dabbed eight to 10 times in multiple locations in the palpebral conjunctiva until it was saturated. The fleece of the sample collector was then placed into the sample transfer window of the test cassette body. The absorbent tip was immersed into the buffer vial for 20 s and laid flat on a horizontal surface for 10 min. Semiquantitative interpretation using color intensity of the result line was done by assigning zero to four points according to the reference image (Figure 1).

### Statistical analysis

Statistical analysis was performed with a commercial program (SPSS for Windows; version 22.0.1; IBM Corp., Armonk, NY, USA). Only right eyes from the age-matched subjects were included for analysis. One-way analysis of variance (ANOVA) and the post-hoc least significant difference test were used to compare continuous variables among and between groups. The Mann–Whitney U and Kruskal–Wallis tests were used when a normal distribution could not be confirmed. Categorical variables between groups were compared using the chi-squared test. Pearson’s correlation analysis was employed to determine the coefficient of correlation between parameters. A *p-*value of less than 0.05 was considered to be statistically significant.

## Results

### Demographics

In total, 195 eyes of 195 patients were analyzed. Of these, group 1 (cyclosporin group) contained 69 eyes, group 2 (diquafosol group) contained 59 eyes, and group 3 (artificial tears eyes) contained 67 eyes. All patients were Korean. The mean age was 59.2 ± 11.5 (range: 23–86) years, and 147 patients (75.4%) were female. Age and gender distribution did not differ significantly between the groups (*p =* 0.375 and 0.901, respectively).

### Conventional dry eye parameters and MMP-9

**Figure 2.**
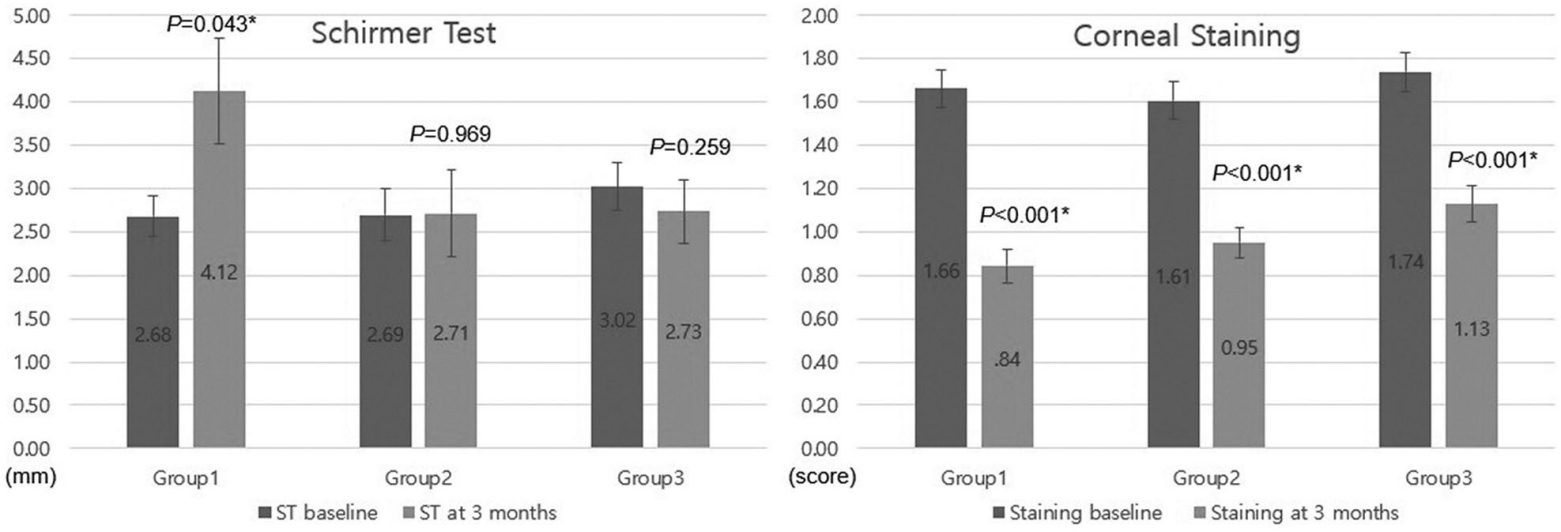
Comparison of Schirmer Test and corneal staining score at baseline and the third month in each group. After three months of treatment, Schirmer I Test improved in group 1 (*p =* 0.043), while corneal staining score improved in all groups (all *p <* 0.001).

Baseline Schirmer I Test and corneal staining scores did not differ between groups (*p =* 0.581 and 0.587, respectively). After three months of treatment, however, corneal staining score had improved in all groups (all *p <* 0.001), while Schirmer I Test only improved in group 1 (*p =* 0.043) (Figure 2).

**Figure 3.**
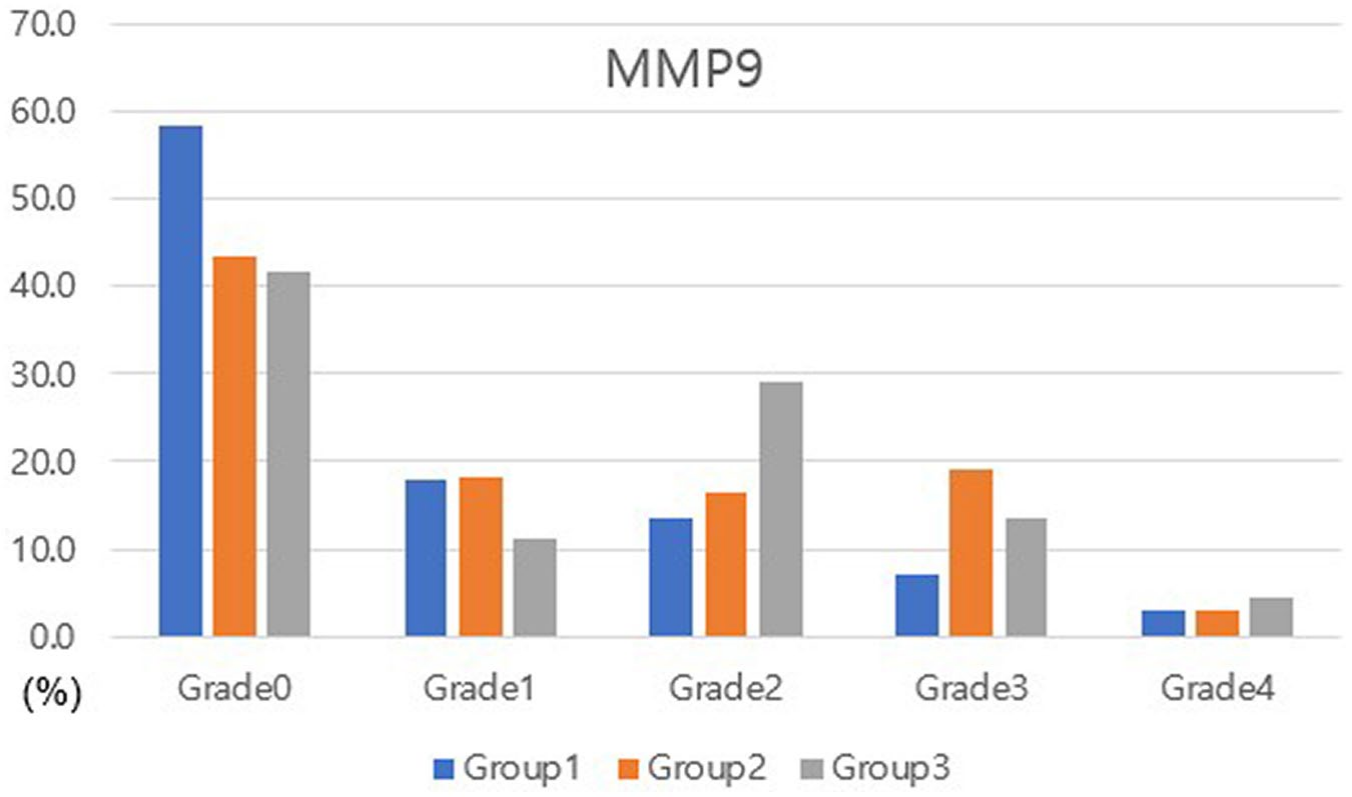
Distribution of MMP-9 grades in each group at the third month. The distribution of MMP-9 grades differed between groups (*p* = 0.002). Th mean MMP-9 grade was lower in group 1 versus in groups 2 and 3 (*p =* 0.008 and 0.001, respectively).

At month three, ANOVA test revealed that corneal staining score, TBUT, and OSDI results differed between the groups (*p =* 0.027, 0.026, and 0.010, respectively). Corneal staining score was greater in group 3 than in group 1 (*p =* 0.008). TBUT and OSDI score were lower in group 3 versus in either groups 1 or 2 (*p =* 0.037 and 0.013 for group 1 and 0.003 and 0.005 for group 2, respectively). MMP-9 expression was significantly lower in group 1 as compared with in groups 2 and 3 (*p =* 0.021 and 0.006, respectively). MMP-9 grade was also lower in group 1 versus in groups 2 and 3 (*p =* 0.008 and 0.001, respectively) (Table 1 and Figure 3).

**Table 1.** Comparison of clinical parameters between groups.

	Group 1 (*n* = 69)	Group 2 (*n* = 59)	Group 3 (*n* = 67)	*p* Value^a^	*p* Value^b^	*p* Value^c^	*p* Value^d^
Baseline							
Age (years), mean ± SD	60 ± 11.2	60.1 ± 11.9	57.7 ± 11.4	0.165	0.965	0.096	0.113
Sex (n), male/female	16/53	16/43	16/51	0.706	0.406	0.757	0.592
Schirmer test (mm), mean ± SD	2.676 ± 2.725	2.692 ± 3.091	3.022 ± 3.259	0.581	0.967	0.404	0.345
Corneal staining (score), mean ± SD	1.662 ± 1.039	1.606 ± 0.908	1.739 ± 1.025	0.587	0.665	0.309	0.525
Month 3							
Schirmer I Test (mm), mean ± SD	4.122 ± 7.180	2.712 ± 5.166	2.731 ± 4.266	0.074	0.058	0.046*	0.979
Corneal staining (score), mean ± SD	0.842 ± .895	0.952 ± 0.729	1.127 ± 0.961	0.027*	0.102	0.008*	0.395
TBUT (sec), mean ± SD	7.270 ± 3.881	7.571 ± 4.520	6.244 ± 3.834	0.026*	0.566	0.037*	0.013*
OSDI (score), mean ± SD	29.2658 ± 21.8996	31.5480 ± 22.2621	37.1961 ± 21.7302	0.010*	0.423	0.003*	0.050*
MMP-9 expression, n (%)	29 (42)	34 (57)	39 (58)	0.012*	0.021*	0.006*	0.819
MMP grade (score), mean ± SD	0.784	1.202	1.276	0.002*	0.008*	0.001*	0.638

SD: standard deviation; TBUT: tear-film break-up test; OSDI: ocular surface disease index; MMP-9: matrix metalloprotease-9.

^a^Comparison between 3 groups.

^b^Comparison between groups 1 and 2.

^c^Comparison between groups 1 and 3.

^d^Comparison between groups 2 and 3.

* Statistically significant *P*-value.

Separately, MMP-9 grade correlated negatively with Schirmer I Test and TBUT and positively with corneal staining score and OSDI score in all study subjects (*p =* 0.005, < 0.001, 0.013, and < 0.001, respectively). In the correlation analysis of each group, these correlations were valid in groups 1 and 3 (*p =* 0.018, < 0.001, 0.015, and < 0.001 for group 1 and 0.001, 0.013, 0.017, and 0.005 for group 3, respectively). In group 2, MMP-9 grade correlated positively with corneal staining and OSDI scores (both *p <* 0.001), but the correlation with ST and TBUT was not statistically significant (*p =* 0.145 and 0.514, respectively) (Table 2).

**Table 2.** Correlation between matrix metalloprotease 9 grade and conventional dry eye parameters.

Group	Schirmer test	Corneal staining	TBUT	OSDI
Total				
Pearson Correlation	−0.144*	0.326*	−0.128*	0.309*
Sig. (two-tailed)	0.005	0.000	0.013	0.000
Group 1				
Pearson Correlation	−0.200*	0.404*	−0.207*	0.300*
Sig. (two-tailed)	0.018	0.000	0.015	0.000
Group 2				
Pearson Correlation	−0.144	0.358*	0.065	0.351*
Sig. (2-tailed)	0.145	0.000	0.514	0.000
Group 3				
Pearson Correlation	−0.288*	0.213*	−0.206*	0.243*
Sig. (two-tailed)	0.001	0.013	0.017	0.005

TBUT: tear-film break-up time; OSDI: Ocular Surface Disease Index.

*Statistically significant *p*-value.

## Discussion

Ocular surface inflammation has been known to be the main pathogenesis of dry eye disease, and treatment options to reduce inflammation have shown effectiveness in this disease [21]. MMP-9 is the most important gelatinase present on the ocular surface [22], and its levels are reported to be higher in the tears of patients with dry eye [4]. In this study, we compared MMP-9 levels between dry eye patients treated with cyclosporin and diquafosol and demonstrated that MMP-9 expression and the level was lower in eyes treated with cyclosporin as compared with diquafosol.

MMP-9 is a molecule strictly related to inflammation, which participates in the physiological and pathologic processes of dry eye. It was reported that MMP-9 accelerates corneal epithelial regeneration in the healing process by modulating the inflammatory response [23]. Hyperosmolarity of dry eye may cause MMP-9 release by the ocular surface cells [2]. In 2013, Sambursky et al. reported a high sensitivity of 85% and specificity of 94% of a point-of-care MMP-9 immunoassay (InflammaDry®) [15]. Elsewhere, the reported prevalence of positive MMP-9 with InflammaDry® ranged from 39% to 50% in dry eye [24–26]. The relatively high detection rate in this study (42%–58%) may be attributable to the nature of the subjects included in the study which only included aqueous-deficient dry eye patients. The report of the research committee of the Dry Eye Workshop concluded that levels of inflammatory factors in tears are increased in aqueous-deficient dry eye, but not necessarily in evaporative type dry eye [27]. Furthermore, a real-time polymerase chain reaction analysis for MMP-9 revealed a discrepancy between dry eye patients with Sjögren’s syndrome and with meibomian gland dysfunction [14]. Although the level was increased in both types compared with control, those with Sjögren’s syndrome showed a more prominent elevation of MMP-9 than those with meibomian gland dysfunction. In this study, we only included dry eyes with aqueous deficiency and, therefore, the detection rate of MMP-9 may have been higher compared with previous studies that included both types of dry eye.

The results of this study revealed that the expression of MMP-9 differed between groups after treatment. It was lower in the cyclosporin group than in the diquafosol group or artificial tears group. In addition, the mean grade of MMP-9 was lower in the cyclosporin group. The increase of MMP-9 activity on the ocular surface can amplify the chronic immune-based inflammation of dry eye [4]. Cyclosporine A has been shown to be an effective dry eye treatment by affecting cytokine levels to control inflammation [28]. One of the suggested mechanisms of action of cyclosporine A in dry eye involves the inhibition of cytokine production and T lymphocytes, resulting in reduced inflammation of the ocular surface and improved tear-film stability [29,30]. Lower expression of MMP-9 after cyclosporin treatment has implications that reduced T lymphocyte activity by cyclosporin may have downregulated the expression of MMP-9 during the vicious inflammation cycle in dry eye [31]. This result is in accordance with a previous study that revealed decreased MMP-9 levels after cyclosporin treatment in patients with keratoconus [32].

In a separate previous study by Messmer et al. [25], MMP-9 level correlated with other dry eye tests including ST, corneal staining, TBUT, and OSDI. In the analysis by group, only TBUT in the diquafosol group did not reveal any significant correlation. There is a controversy regarding whether MMP-9 level correlates with tear-film parameters. While Messmer et al. [25] demonstrated a significant correlation between MMP-9 and TBUT, Schargus et al. [33] reported a discrepancy between TBUT, osmolarity, and MMP-9. A lack of correlation between TBUT and MMP-9 after diquafosol may add uncertainty to this matter, but it is certain that the ocular surface response involving MMP-9 differs between cyclosporin and diquafosol.

An investigation that compared the effect of cyclosporin and diquafosol in dry eye found that Schirmer I Test and conjunctival staining improvement were more prominent in eyes treated with cyclosporin, while TBUT was better in eyes treated with diquafosol [34]. The outcome of this study showed similar results. At the third month, ST was higher and corneal staining score was lower in the cyclosporin group, whereas TBUT was higher in the diquafosol group. The action mechanism of diquafosol is different from that of cyclosporin: specifically, the former is a P2Y2 receptor agonist that activates calcium secretion in the conjunctival epithelium, stabilizing the tear film by enhancing mucin secretion [19]. Because diquafosol does not possess immunomodulatory properties, the apparent difference in MMP-9 expression between the cyclosporin and diquafosol groups can be explained by the fact that the two drugs have different modes of action, along with differences in other parameters.

There are some limitations to the present study, including those inherent to its retrospective design. Additionally, we studied patients with moderate to severe dry eye that met the 2007 International Dry Eye Workshop criteria. The results of our study may therefore not be applicable to all dry eye patients, in particular those with mild symptoms or who require more intensive treatment. Second, MMP-9 baseline values are lacking. Due to the price of the MMP-9 test, it was carried out only once, after treatment, to confirm differences in MMP-9 level between the groups. Nonetheless, baseline parameters were well-matched between the groups, including Schirmer I Test and corneal staining. Third, only TBUT was assessed as a tear-film stability parameter. Additional information on tear osmolarity or tear clearance rate might have helped with analyzing the results of relation between TBUT and MMP-9. Regarding this matter, we measured TBUT using topography to minimize subjective errors. To the best of our knowledge, this is the first study to analyze differences in the effect of cyclosporin and diquafosol on MMP-9 expression that involved a relatively large number of patients with dry eye.

In conclusion, we found that MMP-9 expression and grade were lower after treatment with cyclosporin versus diquafosol in dry eye disease. Anti-inflammatory treatment can decrease ocular MMP-9 levels in dry eye disease. Further research involving a larger spectrum of dry eye types, different ethnicities, and more baseline dry eye parameters are required to validate and expand upon the results of the current study.

## Data Availability

The datasets used and/or analysed during the current study available from the corresponding author on reasonable request.
